# Accumulation of liposomes in metastatic tumor sites is not necessary for anti-cancer drug efficacy

**DOI:** 10.1186/s12967-024-05428-9

**Published:** 2024-07-03

**Authors:** Jessica Kalra, Jennifer Baker, XuXin Sun, Alastair Kyle, Andrew Minchinton, Marcel B. Bally

**Affiliations:** 1 Experimental Therapeutics, BC Cancer Research Institute, Vancouver, BC Canada; 2 Integrative Oncology, BC Cancer Research Institute, Vancouver, BC Canada; 3 Department of Pathology and Laboratory Medicine, University of British Columbia, Vancouver, BC Canada; 4 Faculty of Pharmaceutical Sciences, University of British Columbia, Vancouver, BC Canada; 5 NanoMedicine Innovation Network, University of British Columbia, Vancouver, BC Canada

**Keywords:** Liposomes, Enhanced permeability and retention effect, Metastases/metastasis, Tumor microenvironment, Inter- and intra-tumor heterogeneity, Pre-clinical models, Translational research

## Abstract

**Background:**

The tumor microenvironment is profoundly heterogeneous particularly when comparing sites of metastases. Establishing the extent of this heterogeneity may provide guidance on how best to design lipid-based drug delivery systems to treat metastatic disease. Building on our previous research, the current study employs a murine model of metastatic cancer to explore the distribution of ~ 100 nm liposomes.

**Methods:**

Female NCr nude mice were inoculated with a fluorescently labeled, Her2/neu-positive, trastuzumab-resistant breast cancer cell line, JIMT-1^mkate^, either in the mammary fat pad to create an orthotopic tumor (OT), or via intracardiac injection (IC) to establish tumors throughout the body. Animals were dosed with fluorescent and radio-labeled liposomes. In vivo and ex vivo fluorescent imaging was used to track liposome distribution over a period of 48 h. Liposome distribution in orthotopic tumors was compared to sites of tumor growth that arose following IC injection.

**Results:**

A significant amount of inter-vessel heterogeneity for DiR distribution was observed, with most tumor blood vessels showing little to no presence of the DiR-labelled liposomes. Further, there was limited extravascular distribution of DiR liposomes in the perivascular regions around DiR-positive vessels. While all OT tumors contained at least some DiR-positive vessels, many metastases had very little or none. Despite the apparent limited distribution of liposomes within metastases, two liposomal drug formulations, Irinophore C and Doxil, showed similar efficacy for both the OT and IC JIMT-1^mkate^ models.

**Conclusion:**

These findings suggest that liposomal formulations achieve therapeutic benefits through mechanisms that extend beyond the enhanced permeability and retention effect.

## Background

Previously, our group assessed the heterogeneity of Her2/neu expression and the vasculature within the tumor microenvironment (TME) of tumors that developed at multiple sites following intracardiac injection of Her2/neu positive, trastuzumab-insensitive JIMT-1 cells [1]. When these cells are implanted, they establish tumors that recapitulate some of the intra- and inter-tumor heterogeneity that is observed clinically. Furthermore, these tumors show similarities to metastatic disease in that lesions seeded within different organ sites grow differently despite originating from the same isogenic cell line. We proposed that lipid-based drug delivery systems such as liposomes, can be designed to overcome challenges in the treatment of metastatic tumors with heterogeneous TMEs. Liposomes are customizable drug delivery systems (DDSs) that have the potential to improve the therapeutic index of cytotoxic drugs or drug candidates [2, 3]. There are several liposomal formulations of antineoplastic agents that are approved and being used clinically in the treatment of cancer. Examples include Doxil [4–6], MyoCet [3, 7, 8], and Vyxeos [9–11]. Irinophore C, a liposomal irinotecan formulation developed by our lab [12–16], showed significant promise but was not developed in part due to the prior approval of Onivyde, a liposomal irinotecan formulation that is now being used as second line treatment in patients with pancreatic cancer [17, 18]. Each of these liposomal drugs appear to have advantages over free drugs.

The mechanism of improved efficacy for liposomal formulations is often attributed to passive targeting that results in the accumulation of liposomes in tumors due to the enhanced permeability and retention (EPR) effect [19–22]. The EPR effect is thought to allow nanoparticles within blood vessels to extravasate into tumor tissue because the tumor vasculature is immature and leaky, and the associated lack of a lymphatic system does not allow fluids with liposomes to leave the site [23–27]. However, the role of the EPR effect in improving drug efficacy is controversial [28, 29]. Investigators have reported that the EPR effect is heterogeneous and likely not relevant in all tumors [30, 31]. This may be particularly relevant for metastatic disease, where inter-lesion heterogeneity in drug delivery may limit the benefit of any EPR effect seen in solid tumor preclinical models [32, 33] as well as in the treatment of primary tumors in the clinic [34]. While some investigators are trying to augment or enhance the EPR effect [33] to overcome the challenges of heterogeneous retention, others have reported that the accumulation of liposomal formulations in tumor tissues may be more likely due to active processes such as transcytosis [35, 36], therefore strategically coopting cellular uptake mechanisms through drug design, may be more fruitful in achieving better therapeutic effects. Relevant to our findings, it has also been shown that improved efficacy with liposomal formulations may not be dependent on tumor-specific accumulation at all, but may instead be due to the prolonged circulation lifetime of liposomes and the slow release of active drug cargo over time [37–39]. An effect we have previously demonstrated for liposomal camptothecins in colorectal cancer models [40].

The objective of the experiments conducted in this study was to lay the groundwork for investigating the potential of designing liposomes to address challenges of heterogeneous TMEs. To determine whether there is a relationship between the efficacy of liposomal drugs and the accumulation of liposomes in multiple metastatic sites, in vivo and ex vivo imaging tools were used to measure the distribution of labeled liposomes in both orthotopic tumors (OT) and an intracardiac model of disseminated tumors (IC). OT and IC models were also used to assess the differential efficacy of liposomal doxorubicin (Doxil) and irinotecan (Irinophore C). While our results show improved survival for animals treated with liposomal formulations of the cytotoxic chemotherapies, we found no evidence to suggest that liposomes benefit from any significant accumulation in tumor lesions, suggesting little contribution from the EPR effect. Rather, based on in vivo, ex vivo and multiplex immunohistochemistry (mIHC) imaging of liposome distribution compared to quantitation in the blood compartment, we suggest that the drug released from liposomes and retained in the blood compartment over time may be most relevant to achieving improvements in efficacy. Therefore, to optimize therapy for metastatic disease, we believe that focus should be placed on designing liposomes to extend circulation time and to control drug release.

## Materials and methods

### Cell-lines and culture

JIMT-1 cells were purchased from the German Collection of Microorganisms and Cell Culture (Deutsche Sammlung von Mikroorganismen und Zellkulturen GmbH). Cells were resuspended in freezing media (10% DMS0 in FBS) and slowly frozen in Nalgene^®^ 1 ℃ freezing containers (Rochester NY, USA) containing 100% isopropanol at − 80 ℃ for 24 h before storage in liquid nitrogen. Frozen cells were quickly thawed at 37 ℃, centrifuged to remove freezing media, plated and passaged twice before use in experiments. Cells were maintained in DMEM/high glucose supplemented with L-glutamine (2 mMol/L; DMEM and L-glutamine from Stem Cell Technologies, Vancouver, British Columbia, Canada), 5 mM penicillin/streptomycin (Stem Cell), and 10% fetal bovine serum (FBS) (Hyclone, Logan, UT, USA). All cells were maintained at 37 ℃ and 5% CO_2_ in a humidified atmosphere and allowed to undergo no more than 20 passages. Cells were maintained in the absence of penicillin and streptomycin and screened for mycoplasma prior to preparing the stock of cells that were frozen for future use in animal experiments.

### Lentivirus transfections

JIMT-1^mKate^ cells were generated by lentiviral transfection of JIMT-1 cells with the gene encoding mKate2 [41]. The pFUKW transfer plasmid had been generated by inserting the mKate gene, obtained from the pmKate2-N plasmid (Evrogen), into a pFUW vector backbone [41, 42]. Lentivirus particles were generated by combining pFUKW with the envelope plasmid pVSV-G (Clontech) and the packaging plasmid pDeltaR8.91 in HEK 293 T cells [43]. Viral supernatant was harvested, filtered, and subsequently used to infect target JIMT-1 cells. Resulting JIMT-1^mKate^ cells were sorted by FACS for red fluorescence and the top 12% were used for subsequent experiments.

### Fluorescent liposome preparation

1,2-distearoyl-sn-glycero-3-phosphocholine (DSPC), cholesterol and 1,1 Dioctadecyl-3,3,3′,3,′-Tetramethylindotricarbocyantine Iodide (DiR; DiC_18_(7)) were dissolved in chloroform at a 55:45:0.25 mol ratio. DiR (Thermofisher) is an infrared fluorescent, lipophilic carbocyanine that is photostable when incorporated into the liposomal lipid membrane. The excitation and emission for DiR is in the near infrared (IR) range (750/780 nm). [3H]-CHE was incorporated into the chloroform mixture (5.0–12.5 nanocurie per μmol total liposomal lipid) prior to drying. Chloroform was removed by drying the samples under a steady stream of nitrogen gas until the samples became viscous. The samples were then placed under high vacuum to create a dried lipid film. The dried lipid was hydrated in PBS and the resulting multilamellar liposomes were then extruded 10 times through two stacked 0.1 μm polycarbonate filters using a 10-mL thermobarrel extruder (Extruder^™^, Evonik Transferra Nanosciences, Burnaby, BC, Canada). The extruded unilamellar liposomes had a mean diameter of 110 ± 20 nm as determined by Phase Analysis Light Scattering methods (ZetaPALS, Brookhaven Instruments Corp., Holtsville, NY). The final lipid concentration was determined by measuring [3H] using liquid scintillation counting (LSC) (Packard 1900 TR Liquid Scintillation analyzer). For all in vivo studies, a single liposomal lipid dose of 100 mg/kg was selected. This liposomal lipid dose is (DSPC:Chol) is generally regarded as safe, and is known to be a dose where the pharmacokinetics of the liposomes are not influenced by uptake of liposomes by cells of mononuclear phagocyte system [44]. Studies have shown that “empty” liposomes composed primarily of PC and cholesterol exhibit good safety characteristics and no toxicity has been observed with these formulations, even with repeated administration using doses of greater then 1000 mg liposomal lipid/kg. At this dose the elimination of liposomes (PEG-modified as well as PEG-free liposomes) from the blood compartment follows first-order kinetics within the first 24 h [44]. The use of the non-exchangeable and non-metabolizable [3H]-CHE label (Perkin Elmer Waltham, MA) [45, 46] enabled quantitation of liposome concentration in blood and tissue using LSC, while the near infrared fluorescent, lipophilic, photostable carbocyanine, DiR [47, 48] enabled in vivo fluorescence imaging (IVFI) tracking of the liposome in the live animal as well as multiplex immunohistochemistry (mIHC) in tissue sections.

### Animal studies

All animal studies were conducted in accordance with institutional (University of British Columbia) guidelines for humane animal treatment and according to the current guidelines of the Canadian Council of Animal Care. Mice were maintained at 22 ℃ in a 12-h light and dark cycle with ad libitum access to water and food. The studies described herein, used female NCr nude mice weighing between 18 and 25 g which were obtained from Taconic (Oxnard, CA, USA) and maintained in an SPF-Facility. NCr nude mice were selected because the tumor models derived following injection of JIMT-1 human breast cancer cells require the use of an immune compromised mouse. The JIMT-1 cells consistently develop into tumors following IC and OT inoculation in this strain of mice [1]. For comparison purposes studies completed in tumor free mice were also done using the NCr mice. It should be noted that in our experience “empty” liposomes administered at 100 mg/kg exhibit similar pharmacokinetics and biodistribution in immune-competent and immune-compromised mice. Animals were housed in groups of 4 or 5. Long-term survival was determined based on the time in days when mice were terminated due to tumor ulceration, the presence of tumors exhibiting volumes in excess of 800 mg, and/or signs of deteriorating animal health requiring euthanasia, as defined by a health monitoring standard operating procedure.

To initiate orthotopic (OT) disease, 2 × 10^6^ JIMT-1^mkate^ cells were inoculated into the mammary fat pad in a volume of 50 µL media using a 28-gauge needle. The method to start systemic disease had been previously described [49]. Briefly, for the intracardiac (IC) inoculation of cells, animals were anesthetized using isoflurane and positioned so that a 26-gauge needle attached to a 1 mL tuberculin syringe could be inserted at a 30-degree angle immediately caudal to the xyphoid process. 1.8 × 10^5^ JIMT-1^mkate^ cells in a volume of 100 µL of media were slowly (over 30 to 60 s) injected into the left ventricle. Animals were monitored for tumor growth, body weight and health status. Tumor growth for both OT and IC models, was monitored using the Maestro imaging system (Perkin Elmer, MA, USA) as described below. Animal health status was monitored daily, and the humane endpoint was determined based on the PICOR clinical scoring system. The major humane endpoint indications were laboured breathing and signs of pain. Animal body weight was measured every Monday and Friday.

### Efficacy study

36 animals were randomized into 2 groups of 18 according to the site of tumor cell inoculation (OT or IC) and further randomized into 3 subgroups of 6; a saline treatment group, a Doxil treatment group (Taro Pharmaceuticals Inc., Ontario, Canada) and an Irinophore C treatment group (produced in house [50]). NCr mice bearing tumors were established as described above using the parental JIMT-1 cell line, and animals were monitored for signs of established disease. For the OT tumor model, tumor growth was assessed using caliper measurements. On day 8, animals were treated intravenously (iv) with saline, Doxil (1 mg/kg; Q14Dx2), or Irinophore C (20 mg/kg; Q4Dx3). The liposomal lipid dose was adjusted to 100 mg/kg—the liposomal lipid dose used when administering the DiR labelled liposomes. For Irinophore C, the drug to lipid (D/L) ratio of this formulation was 0.2 (mol/mol) and the lipid dose was calculated to be 108 mg/kg at the 20 mg/kg irinotecan dose. For Doxil, drug-free liposomes (hydrogenated soybean PC (HSPC), Chol, and 1,2-distearoyl-sn-glycero-3-phosphoethanolamine- N-[methoxy(polyethylene glycol)-2000] (DSPE-MPEG2000; (weight ratio of 3:1:1) were prepared as described above. These empty liposomes were then added to the Doxil formulations to achieve a final liposomal lipid dose of 100 mg/kg lipid and 1 mg/kg doxorubicin. Post injection, animals were monitored for tumor growth (caliper measurements for OT tumors and IVFI for IC tumors), changes in body weight and survival.

### Maestro imaging

Imaging was performed once per week to monitor tumor growth and localize sites of metastasis. Fluorescent images of the whole mouse body were obtained using the Maestro in vivo fluorescence imaging system (Perkin Elmer, MA, USA). Mice were anaesthetized using isoflurane and fluorescent images were captured under the following conditions: 1. To identify the location of mKate (the tumor cells), the “Green” filter model was used; excitation filter range 523 nm (503–548 nm); emission filter range 560 nm longpass; acquisition setting; 560–750 nm in 10 nm steps; and 2. To image DiR distribution, the “NIR “ filter model was used; excitation filter range 704 nm (684–729 nm); emission filter range 745 nm longpass; acquisition setting; 740–950 nm in 10 nm steps. Auto-fluorescence of mice, mKate and DiR fluorescence was obtained and then unmixed using the Maestro spectral software. Ex vivo imaging of mKate and DiR in the liver, lungs, kidney, adrenal glands, ovaries and brain was performed for each animal.

### Pharmacokinetics

20 non-tumor bearing mice, 20 mice with established OT tumors and 30 mice with tumors that developed following IC tumor cell injection (n = 4 to 6 per time point) were individually weighed and injected i.v. with DiR labelled liposomes. The animals assigned to the 48-h time-point were imaged 1, 4, 8, 24, and 48 h post injection using the maestro in vivo imaging system. Animals were terminated at the designated time-point by isoflurane followed by CO_2_ inhalation. Upon last breath, animals were removed from the inhalation chamber and approximately 500 μL of blood was collected by cardiac puncture with a 25G needle and the blood was placed into the appropriate EDTA containing microtainer tubes. Blood was stored on icse until plasma was separated by centrifuging the blood samples at 2500 rpm for 15 min. The plasma was then pipetted off and placed into labeled vials. Plasma was stored at – 80 ℃. OT tumors and organs/tissues with visible tumors were harvested. Harvested tissues were divided into two whenever possible. One half was collected in pre-weighed scintillation vials for further analysis; the second half was placed in OCT and frozen immediately on dry ice. Once harvested all blood samples and OCT samples were stored at − 80 ℃. Blood samples and tissue samples collected in scintillation vials were processed to obtain [3H]-CHE. OCT samples were sectioned and used for mIHC analysis.

### Liquid scintillation counting (LSC)

100 µL of plasma was added to 5 ml scintillation fluid and the amount of [3H] present was measured by LSC (Packard 1900TR Liquid Scintillation Analyzer). DPMs were converted to liposomal lipid concentration per 1 mL of plasma using the specific activity of the injected DiR labelled liposomes. Tumors or tissues containing tumors were harvested and the presence of mKate positive cells was confirmed by IVFI. Tumors or tissues with confirmed evidence of tumor growth were harvested. Harvested tissue was weighed and then solubilized using 500 µL of Solvable (Perkin Elmer, Boston, MA) and incubated overnight at 50 ℃. Organ homogenates were decolorized using 200 µL H_2_O_2_ and 50 µL EDTA and subsequently incubated overnight at room temperature. The samples were assessed for [3H]-CHE using LSC, and these data were then used to estimate the amount of DiR labelled liposomes per mg of tissue.

### Tissue collection and immunofluorescence

Animals were euthanized by first anesthetizing them with isoflurane followed by CO_2_ asphyxiation, tumors and organs were excised, cut in half in the sagittal plane, and one half embedded in OCT and immediately placed on dry ice. Samples were stored at − 80 ℃. Ten, 10 μm tumor cryosections were cut using a Cyrostar HM560 (Microm International, Waldorf, Germany), air-dried, and imaged for exogenous marker native fluorescence; mKate (visualized at 633 nm) and DiR (visualized at 750 nM). Sections were fixed in 50% (v/v) acetone/methanol for 10 min at room temperature and subsequently stained for: 1. CD31 using a rat monoclonal anti-mouse CD31 antibody (BD Pharmingen) and Alexa 647 secondary antibody (Invitrogen); 2. Her2/neu using a monoclonal anti-human secondary antibody tagged with Alexa 546 (Invitrogen); and 3. Cell nuclei using Hoechst 33,342, Bis-Benzimide (Sigma) (8 μg/mL at 37 ℃) for 30 min.

### Image acquisition and analysis

The imaging system consists of a robotic fluorescence microscope (Zeiss Imager Z1), a cooled, monochrome CCD camera (Retiga 4000R, QImaging), a motorized slide loader and x–y stage (Ludl Electronic Products), and customized NIH-ImageJ software described in detail elsewhere [51, 52]. The system allows adjacent microscope fields of view to be imaged and automatically tiled to produce a montage of the entire tumor cryosections at a resolution of 0.75 μm/pixel for qualitative and quantitative analysis. All variables stained on the same section were imaged separately using the monochrome camera and subsequently the ImageJ software application (NIH ImageJ) was used to overlay and align each image for analysis and to generate false-color images. Images were screened manually to identify regions of interest (ROI) (i.e. tumor tissue versus normal tissue) and artifacts of processing.

### Statistical analyses

Data was plotted and analyzed using Prism 9 version 9.5.1. Area under the curve (AUC) was calculated using the trapezoid rule. Prism computes median survival times (MST) from Kaplan–Meir curves as the time at which the curve crosses 50% survival. Differences were considered statistically significant at p < 0.05.

## Results

### In vivo distribution of DiR labelled DSPC/Chol liposomes in tumor-free animals

**Fig. 1:**
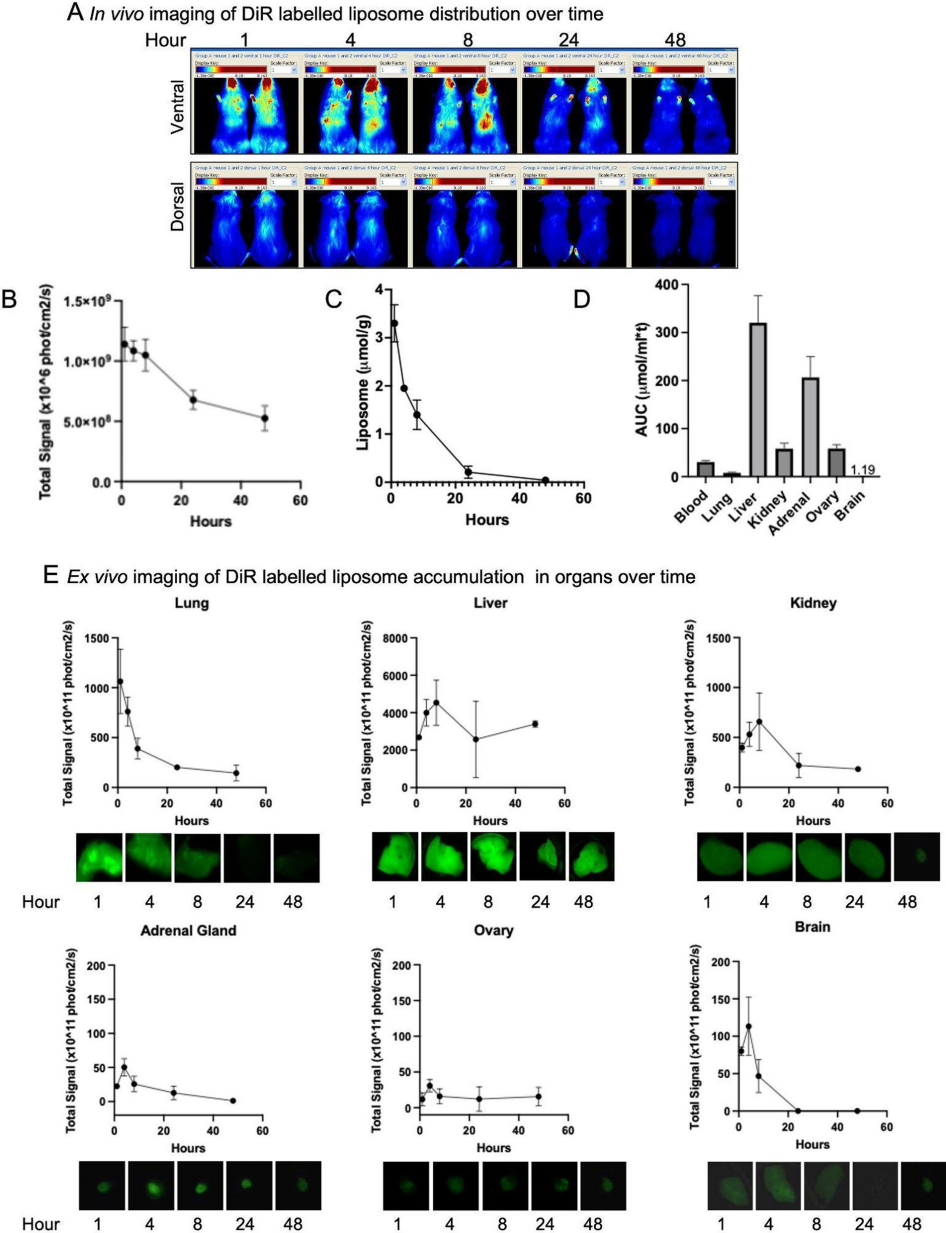
Tumor naïve mice were treated with a single i.v. injection of DiR labelled liposomes at a lipid dose of 100 mg/kg. In vivo fluorescence imaging of the distribution of DiR over 48 h is shown (**A**). Fluorescence signal intensity in the whole animal was quantified (**B**) and demonstrated to have decreased by 58%, 48 h post injection. DiR labelled liposome levels following injection was measured using liquid scintillation counting ([3H]-CHE) and the plasma elimination (**C**) and AUC_0-48Hr_ for each organ was calculated (**D**). Ex vivo imaging of tumor naïve organs and related fluorescence signal intensity over 48 h is shown (**E**)

Dual-labelled liposomes were prepared with both radioactive ([3H]-CHE) and fluorescent (DiOC18 (7); ‘DiR’) markers in order to be able to assess the fate of the liposomes after administration using in vivo fluorescent imaging (IVFI) as well as liquid scintillation counting (LSC). To optimize in vivo imaging of the DiR labelled liposomes, initial studies focussed on tumor free mice injected i.v. with a well-tolerated liposomal lipid dose of 100 mg/kg (see Methods). These animals were subsequently imaged at 1, 4, 8, 24 and 48 h post injection using the Maestro IVFI system. Visual inspection of the images suggest that DiR fluorescence was distributed throughout the animal at 1 h but dissipated over 48 h. Whole body fluorescence is shown (Fig. 1A). Images were quantified by plotting photons/cm/sec at each time point. The results (Fig. 1B) indicate that there was loss of signal intensity as a function of time after injection. Relative to the 1 h time point, there was approximately 60% loss of signal intensity at 48 h. In contrast, analysis of [3H]–CHE using LSC demonstrated greater than 90% loss of liposomal lipid from the plasma compartment 24 h after liposome injection (Fig. 1C). It is important to note that the whole-body fluorescence imaging reflects liposomes in the blood compartment as well as liposomes that have localized in tissues/organs. Although the images in Fig. 1A suggest that liposomes may be accumulating in the animal’s head, this is not due to liposomes entering the brain. Liposomes cannot cross the blood–brain barrier. DiR labelled liposomes or liposomes labelled with other fluorescent dyes have been used in biodistribution studies [53–55]. In each of these studies, fluorescent signal was observed in the region of the head (mainly the nose, mouth and ears) and the fluorescent signal decreased over time. This is consistent with our observations. We believe that the fluorescence observed in the cranial region at earlier time points is likely coming from liposomes in the vasculature and blood vessels near the skin. This interpretation is supported by previous studies as well as images of isolated tissues and organs. It is worth noting that the high fluorescent signal in the head is only shown on the ventral side and not the dorsal side of the animal. This is because, compared to the dorsal side, the ventral side has a higher blood supply to the nose and mouth where the highly vascularized areas used for sensing are found [56]. Furthermore, because we are using a high lipid dose (100 mg/kg), we expect that the reticuloendothelial system is fully saturated and the liposomes will remain in the circulation for a longer period of time [44]. This means that any highly vascularized region of the animal, such as the area near the nose and mouth, will show strong signal for longer. The limitation of IVFI is that it does not allow us to differentiate between signal that is confined in blood vessels or signal that is diffused into the tissue. This has to do with the poor tissue resolution of the modality [57, 58]. For this reason, both ex vivo and mIHC imaging of the tissue was also completed. As shown in Fig. 1D, there is very little detectable fluorescence in the brain. Furthermore, as shown in Fig. 1E (Brain), the data demonstrates that the levels of fluorescence detected in the brain decrease at later time points. Together this data suggests that the fluorescence detected in the brain is most likely due to the brain's blood volume and the fluorescent liposomes within the blood. LSC measurements of radioactivity are also included as a comparison as this measurement represents liposomes exclusively in the serum.

To measure the distribution of DiR labelled liposomes in organs, the lung, liver, kidney, adrenal glands, ovaries and brain were harvested from each animal (n = 6) and imaged ex vivo or prepared for LSC of [3H]–CHE (see Methods). The selection of organs examined was based on previous studies which showed that tumors consistently developed in these sites following intracardiac injection of JIMT-1 cells [1]. The AUC_0–48 h_ was calculated for each organ collected from animals injected with the [3H]–CHE/DiR labelled liposomes (Fig. 1D). The data suggests that there was significant liposomal lipid accumulation in the liver and, surprisingly, in the adrenal glands over 48 h. Furthermore, while the level of [3H]-CHE in the lung, liver, kidney, brain and plasma decreased over time, the levels in the adrenal glands and ovaries remained constant (data not shown).

Fluorescent images of excised organs (Fig. 1E) show that DiR fluorescence in the lung decreased following administration at a rate that was comparable to that seen in the whole animal as well as to the plasma elimination results. However, in other organs the fluorescence images show accumulation of the liposomes, reaching a peak intensity at 4 (adrenal gland, ovary, and brain) to 12 (liver and kidney) hours. All organs other than the liver showed dissipation of the signal at 48 h. The fluorescence intensity observed in the ovary, adrenal gland and brain was weak relative to that seen in the whole body, lung and liver. The imaging results for the liver suggest that the fluorescence is maintained, even though the quantitation of [3H]-CHE indicates loss of the liposomes over time. This specific result suggests that the fluorescent lipid DiR may be metabolized in the liver over 48 h and a fluorescent metabolite is retained in the tissue. Therefore, fluorescence at later time points may not be reflective of the liposomal formulation, but rather a metabolite of the DiR-lipid.

### In vivo distribution of DiR labelled liposomes in animals bearing OT and IC JIMT-1 tumors

**Fig. 2:**
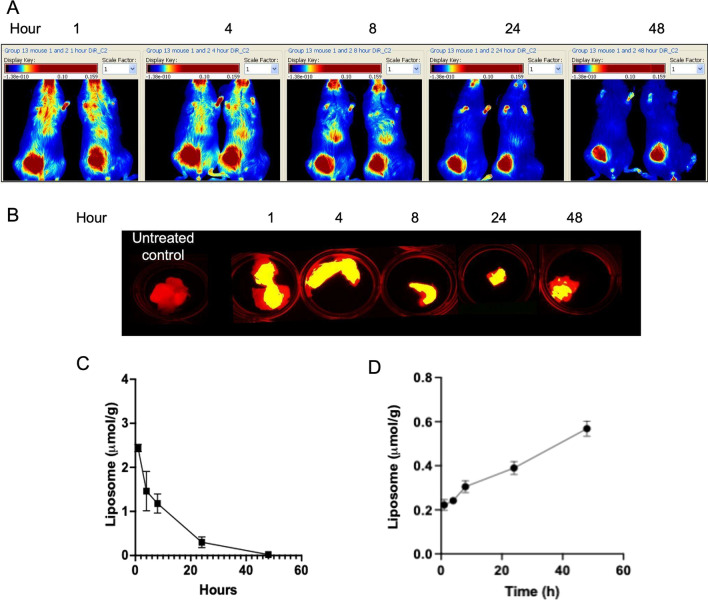
Mice bearing orthotopic (OT) JIMT-1^mKate^ tumors were given a single i.v. injection of DiR labelled liposomes at a liposomal lipid dose of 100 mg/kg. In vivo fluorescence imaging of DiR was performed at 1, 4, 8, 24 and 48 h. Representative images are shown (**A**). Ex vivo imaging of DiR in the OT JIMT-1 tumors at 1, 4, 8, 24 and 48 h is shown (**B**). Plasma elimination (**C**) and OT tumor accumulation (**D**) of injected DiR labelled liposomes in mice with established JIMT-1 OT tumor bearing animals was measured using liquid scintillation counting

JIMT-1^mkate^ cells were inoculated into the mammary fat pad of female NCR mice to establish OT tumors (see Methods). Tumor progression was monitored using IVFI over the course of 28 days. At this time the tumors reached an average of size of 500 mm^3^ (measured by caliper; approximately 500 mg). Once OT tumors were established, the mice were injected (i.v.) with DiR labelled liposomes and IVFI was used to assess DiR distribution at 1, 4, 8, 24 and 48 h after injection. Representative images are provided in Fig. 2A. Inspection of the in vivo images indicates that the whole-body fluorescent signal following injection of DiR labelled liposomes dissipated over 48 h, while the DiR signal intensity in the OT tumor was retained over time. Representative maestro images of excised tumors collected 1, 4, 8, 24 and 48 h after injection of the DiR labelled liposomes are provided in Fig. 2B. Co-localization (yellow) of mKate fluorescence (588 nm; red; tumor) with DiR fluorescence (750; green; liposomes) is observed. Plasma elimination of the DiR labelled liposomes (Fig. 2C, as measured by the liposomal lipid tag [3H]-CHE) in mice with established OT tumors was comparable to that observed in tumor free animals (Fig. 1C). The liposomal lipid AUC_0–48 h_ was 23 μmol/ml*hr in the OT tumor bearing mice and 30 μmole/ml*hr in the control, non-tumor bearing, mice. Liposome concentrations were quantified in OT tumors using [3H]-CHE as the liposomal lipid label (Fig. 2D). Results show that there was accumulation of liposomes in the OT tumors over 48 h. Based on the liposomal lipid dose of 100 mg/kg and the [3H]-CHE accumulation data, it can be calculated that 17.4% of the injected liposomes were distributed to the OT tumors at 48 h.

**Fig. 3:**
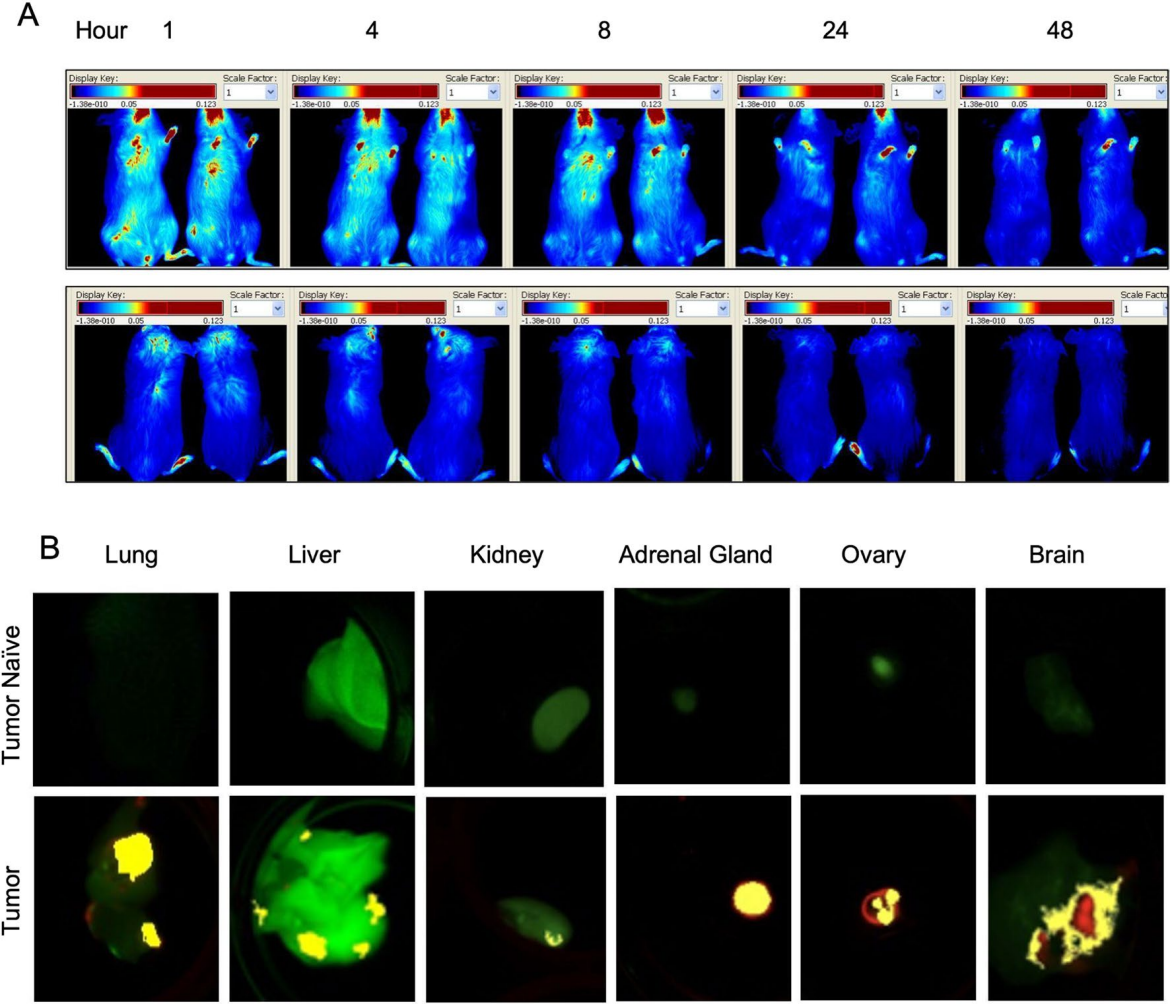
Mice bearing tumors arising following IC injection of JIMT-1^mKate^ cells were given a single i.v. injection of DiR labelled liposomes at a liposomal lipid dose of 100 mg/kg. In vivo fluorescence imaging of DiR was performed at 1, 4, 8, 24 and 48 h. Representative images are shown (**A**). Ex vivo imaging of DiR (green) in JIMT-1^mKate^ tumor positive (red) lung, liver, kidney, adrenal gland, ovary and brain, 24 h post injection (bottom panel) are compared to tumor naive organs (top panel) (**B**). Co-localization (yellow) of DiR and mKate was observed

JIMT-1^mkate^ cells were injected into the left ventricle of animals to seed tumors systemically. Animals were subsequently imaged twice weekly using IVFI to assess tumor development throughout the body (see Methods and reference 1; [1]). Similar to previous studies, mice consistently developed JIMT-1^mkate^ tumors in the lung (6 animals), liver (6), kidney (3), ovary (6), adrenal gland (6) and brain (4). Once established, mice bearing tumors were injected i.v. with DiR labelled liposomes (100 mg/kg) and IVFI was performed at 1, 4, 8, 24 and 48 h post injection (Fig. 3A). Systemic levels of DiR labelled liposomes dissipated over 48 h as expected from the results in Fig. 1A and B. Representative images of tumor naïve lung, liver, kidney, adrenal gland, ovary and brain are provided alongside JIMT-1^mkate^ positive organs excised 24 h after DiR labelled liposome injection (Fig. 3B). In tumor positive organs, colocalization (yellow) of DiR labelled liposomes (green) within some tumors (red) can be observed. The tumor accumulation of liposomal lipid was measured in the orthotopic model using [3H]-CHE as a non-exchangeable, non-metabolizable liposomal lipid marker (Fig. 2B and D). The images in 2B show the florescence, but the results in 2D are based on the use of [3H]-CHE. It was relatively easy to do this because the tumors implanted orthotopically grew consistently and were easy to isolate from surrounding tissue. When using the metastatic model two problems arose. First, it was difficult/impossible to isolate tumors that were within the tissue. Thus, quantification would have represented liposome accumulation in the tissue as well as the tumor, not the tumor alone. For example, the normal liver tissue is known to accumulate liposomes, but in a liver that has established tumors it becomes very difficult to distinguish between liposomes in the normal liver and liposomes in the tumor. The second issue was the inherent variability in tumor seeding in the metastatic model. This variability occurred both between animals (throughout the body) and within individual organs. So, while the intracardiac model was typically initiated using 6 to 10 mice per group and all mice developed metastatic disease, not all mice developed liver metastases, or lung metastases, etc. This variability and the fact that the tumors within the tissue could not be isolated meant that quantification was not possible. It is important to clarify that the primary purpose of the imaging in this study was qualitative analysis. IVFI was used to visualize the presence of tumors and the distribution of the liposomes, but we could not define a way to quantify tumor delivery of the liposomes in tumors arising in multiple tissues/sites.

**Fig. 4:**
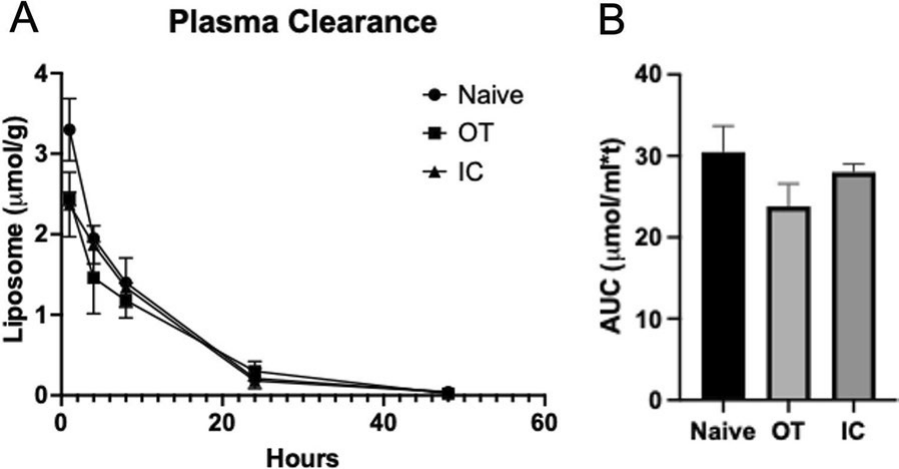
Mice with mKate positive tumors following IC injection JIMT-1^mKate^ cells were given a single i.v. injection of DiR labelled liposomes at a liposomal lipid dose of 100 mg/kg. Plasma elimination (**A**) and the estimated AUC_0-48Hr_ (**B**) of the liposomes from control (non-tumor bearing) mice, mice with OT JIMT-1^mKate^ tumors and mice with IC JIMT-1^mKate^ tumors are compared

As indicated in Figs. 1 and 2, the plasma elimination pattern of liposomes in control mice and mice bearing established OT JIMT-1^mkate^ tumors was comparable and this is consistent with the plasma elimination of liposomes in mice bearing IC JIMT-1^mkate^ tumors (Fig. 4A and B). The AUC_0–48_ for mice with IC injected JIMT-1^mkate^ cells was 28.66 μmol/ml*hr.

### Intra- and inter-metastatic heterogeneity of tumor vasculature and DiR-labelled liposome distribution

To perform mIHC evaluation of the distribution of liposomes in OT tumors as well as tumor naïve and tumor bearing lung, liver, kidney, adrenal glands, ovaries and brain, tissues were harvested from animals 1, 4, 8, 24 and 48 h post injection of DiR labelled liposomes. Collected tissues were frozen and cryo-sectioned for imaging of mKate to determine presence of cancer cells and DiR to determine liposome distribution. Further immunohistochemical staining was completed for Her2/neu and blood vessels (CD31).

**Fig. 5:**
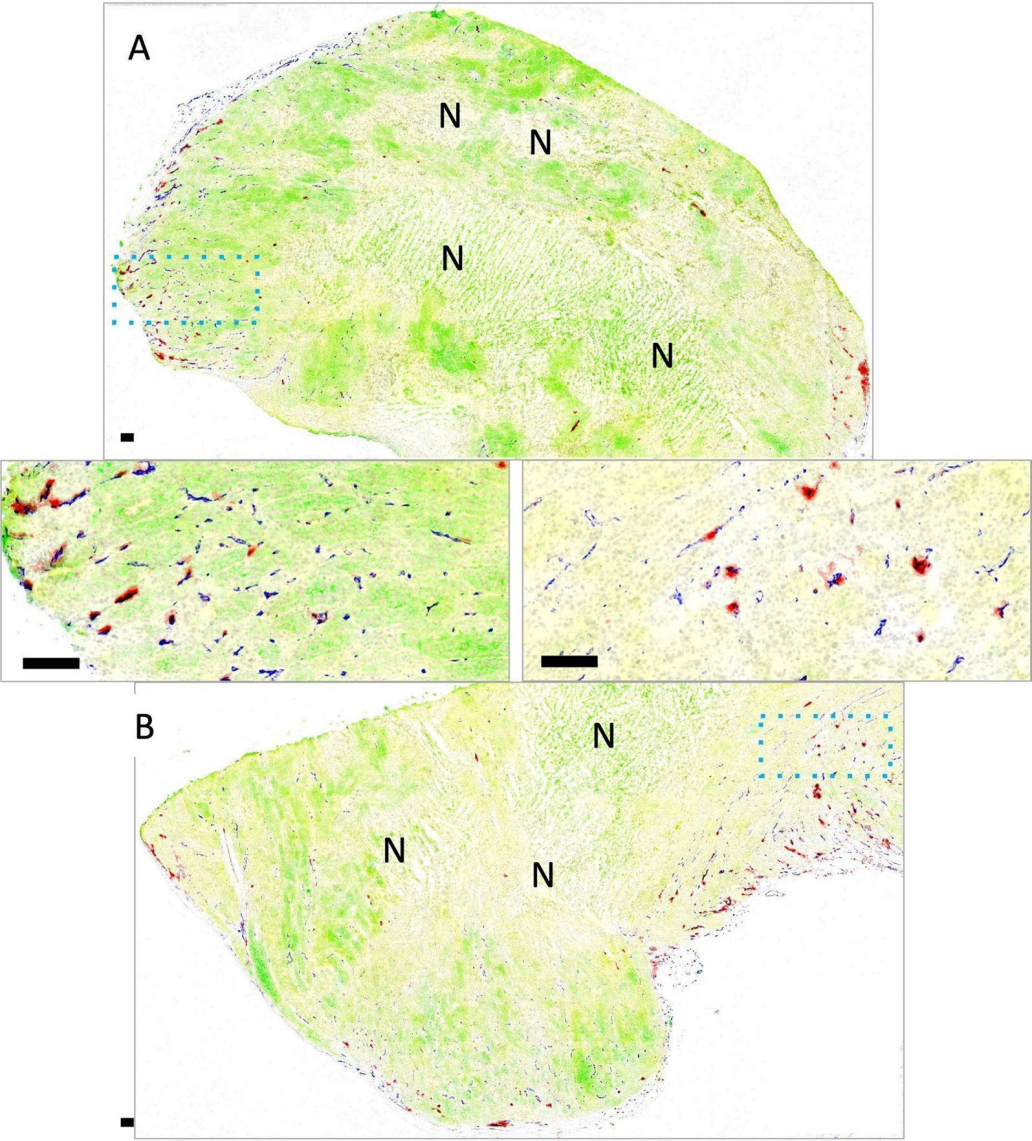
Tumors grown orthotopically in the mammary fat pad are shown with mKate (yellow) and Her2/neu expression (green) overlayed on Hoechst nuclear dye (grey), with CD31 immunostaining (blue) and DiR fluorescence (red; overlapped with CD31 in black). Tumor A (top) was excised 8 h following DiR administration, and tumor B (bottom) at 24 h. Both tumors show heterogeneous Her2/neu and mkate with some necrotic areas (marked with N). Insets illustrate the high degree of inter-vessel heterogeneity, where DiR colocalizes with only a select proportion of CD31 positive blood vessels, often with neighbouring vessels showing alternate patterns. No significant increase in extravascular distribution of DiR is noticeable at the longer 24 h time-point (**B**) relative to 8 h (**A**). Scale bars = 150 µm

Multiplex IHC images of OT tumors illustrate the limited distribution of DiR labelled liposomes (red) into the tumor tissue 8 h (Fig. 5A) and 24 h (Fig. 5B) post injection. DiR is most frequently observed around CD31-labeled vascular structures (blue). Significant inter-vessel heterogeneity is observed, with extravasated DiR labelled liposomes seen around some vessels and none at others, even at the timepoints (8 and 24 h) where relatively high accumulation of liposomes was indicated using IVFI, and LSC. Nuclei are shown in gray, overlayed by mKate (yellow) and Her2/neu (green). Multiplex IHC demonstrates heterogeneous expression of these markers in the orthotopic tumor model as previously described [1]. Areas of necrosis are also identified (**N**).

**Fig. 6:**
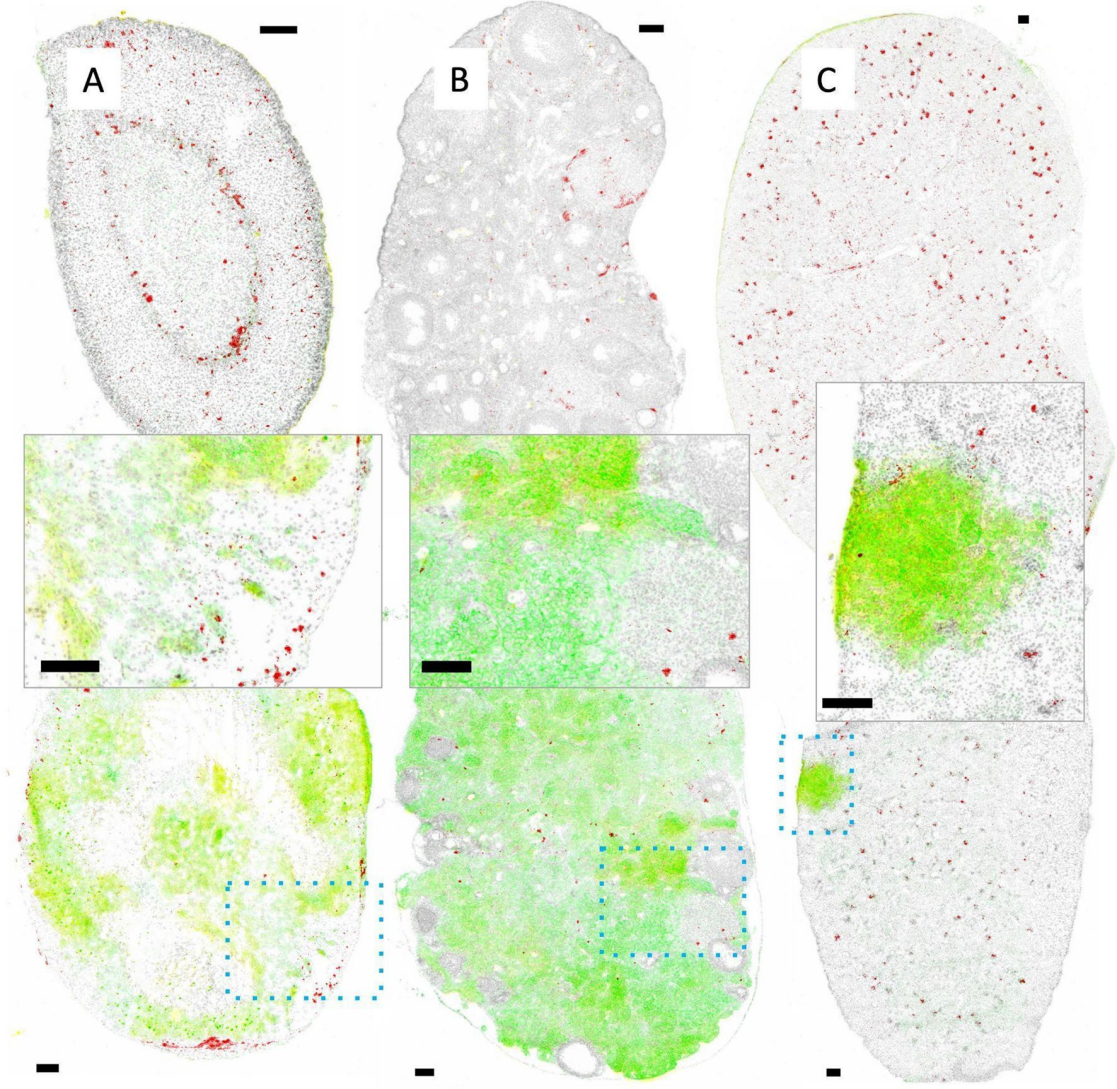
Representative whole adrenal gland (left), ovary (middle) and kidney (right) from non-tumor bearing animals (top) compared with organs bearing metastatic lesions (bottom; insets with enlarged views) are shown. Tumor tissue is identified via mKate fluorescent signal (yellow) and immunostaining of Her2/neu (green); either mKate or Her2/neu positivity suggests tumor growth. Both Her2/neu and mKate expression show heterogeneity, requiring visualization of both markers in addition to pathological review of the tissue in order to identify tumor sites. DiR (red) and nuclear dye Hoechst 33342 (grey) are also included in the image overlays. Organs such as the adrenal gland and ovary are largely overtaken with tumor growth, whereas the kidneys were more likely to have discrete lesions as shown. Liposomal DiR is seen in a few isolated spots of adrenal glands and ovaries, without additional uptake in the tumor-bearing sites within the organs. As expected, the glomeruli of the kidney show greatest DiR signal distributed throughout the organ, with little to no uptake observed within the Her2/neu and mKate positive tumor region. Scale bars = 150 µm

**Fig. 7:**
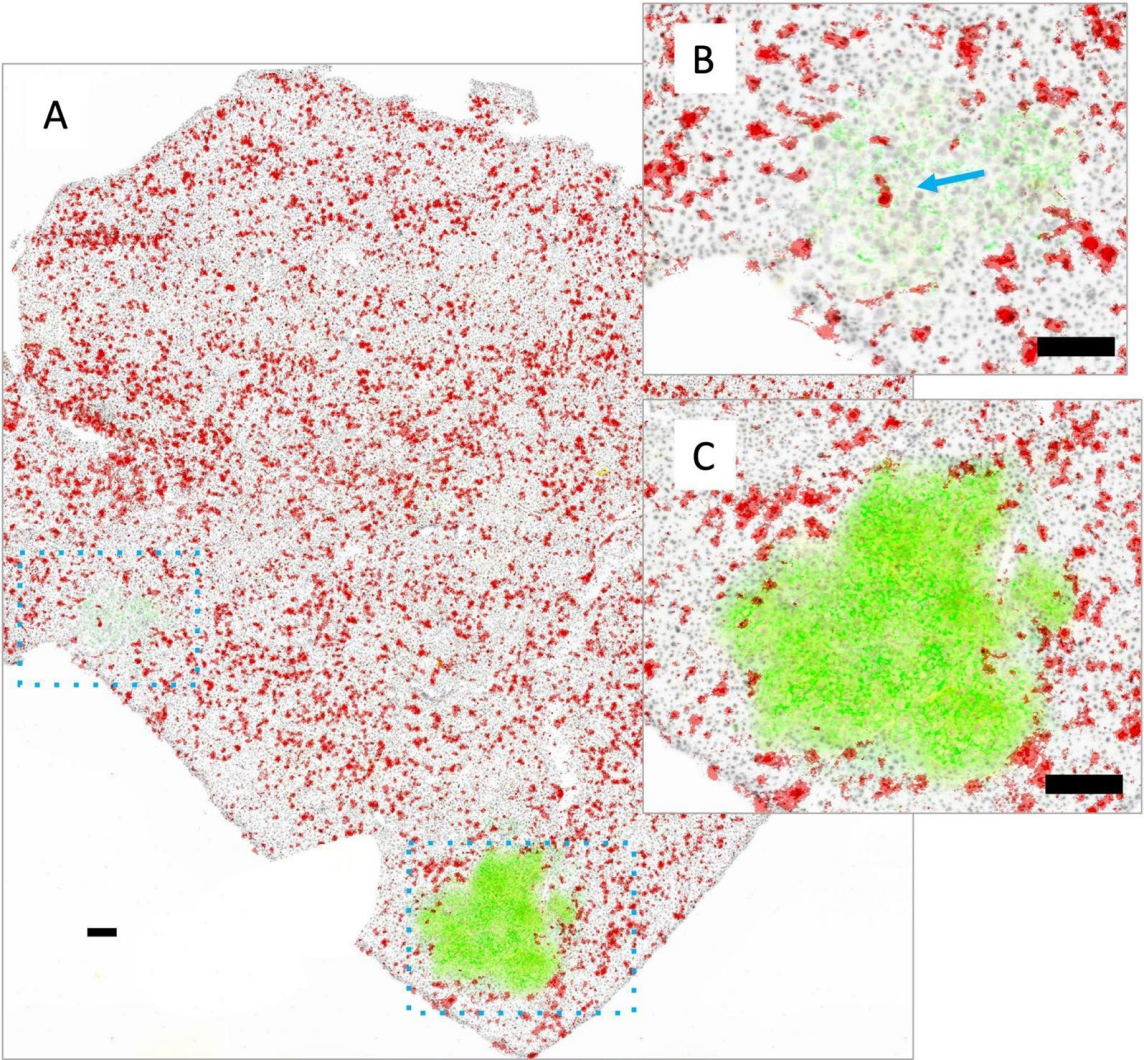
Metastatic tumor nodules in the liver grow as discrete nodules as shown in a representative image of one liver section (**A**). The metastatic lesions are heterogeneous in their degree of Her2/neu (green) and mKate (yellow) expression. Shown in B is one nodule with little to no Her2/neu or mKate and in C another nodule with both markers. DiR fluorescence (red) is evenly distributed throughout the liver, while tumor lesions are largely zones of exclusion for the liposome. One nodule has some central DiR positivity (**B**, blue arrow) while the other has possible DiR positivity at the margins of normal tissue (**C**). Scale bars = 150 µm

**Fig. 8:**
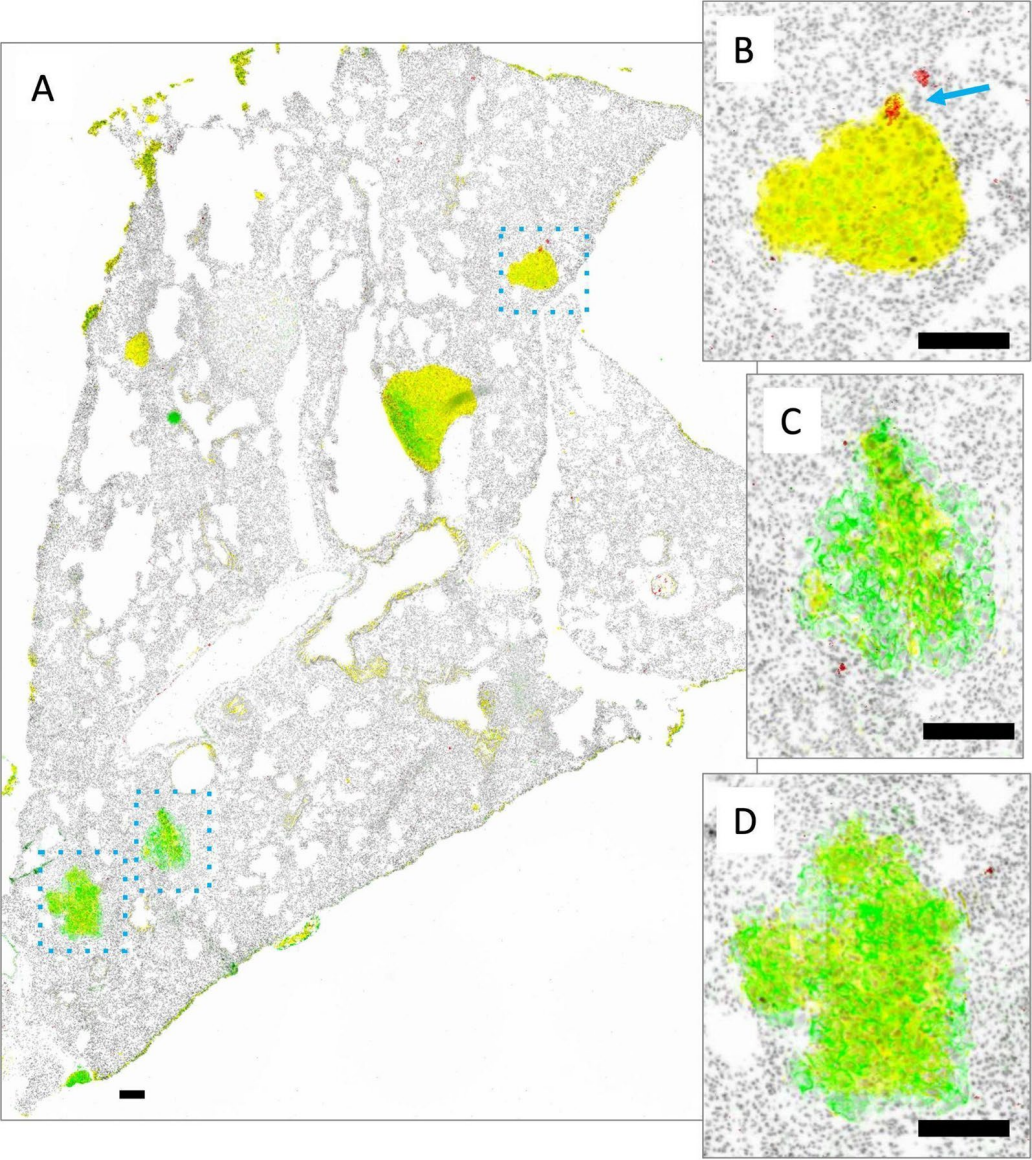
Metastatic tumor nodules in the lung grow as discrete nodules within the organ as shown in a representative image of one lung section (**A**). The metastatic lesions all show mKate (yellow; high for all 3 nodules shown) but are heterogeneous in their degree of Her2/neu expression (green; low for **B**; high for **C**, **D**). DiR fluorescence (red) is generally very low in the lung. Except for two locations adjacent to a single nodule (**B**; blue arrow), no other nodules show DiR positivity. Scale bars = 150 µm

**Fig. 9:**
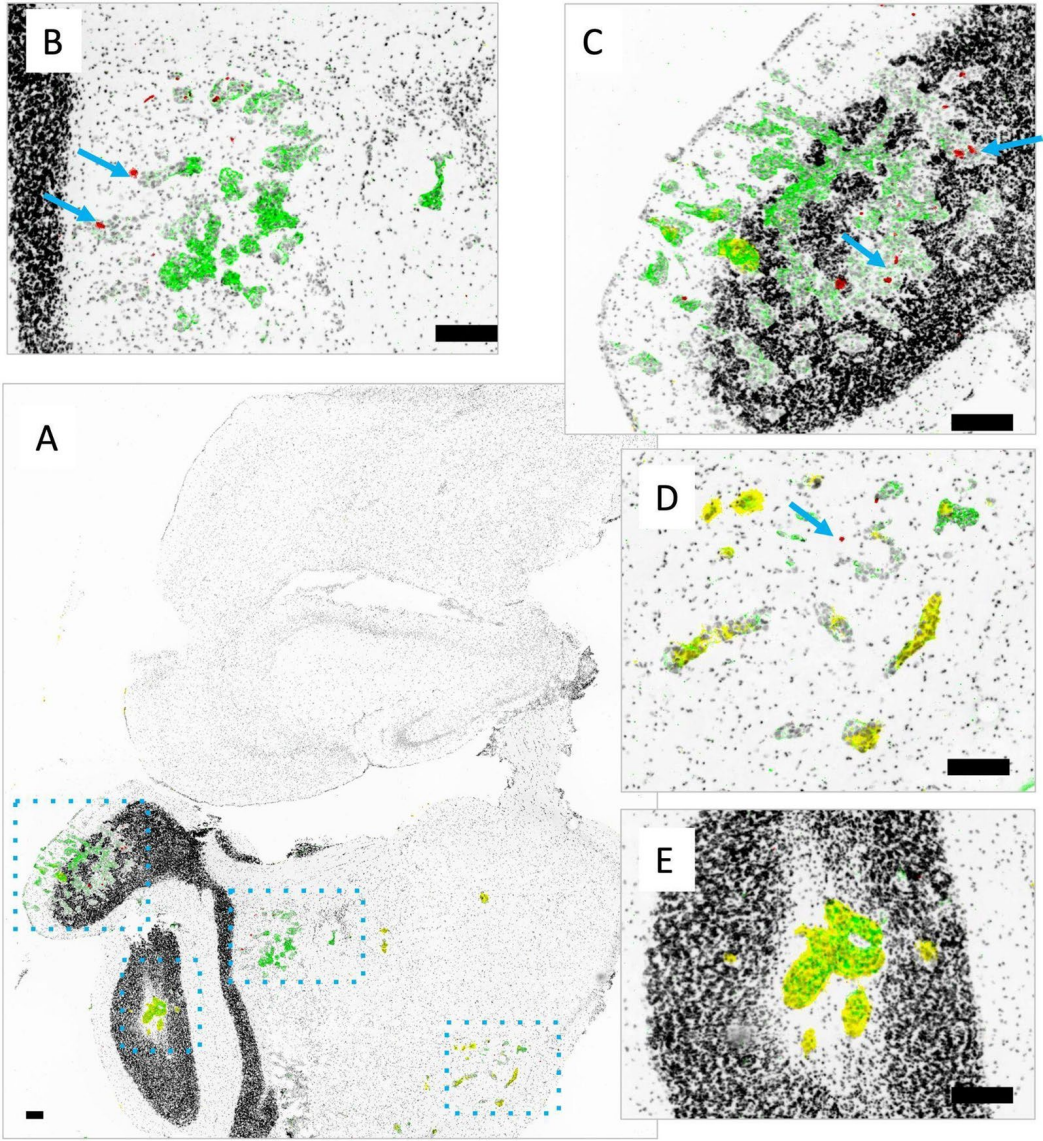
Metastatic tumor nodules in the brain grow as small clusters of cells throughout a whole brain as seen in the representative image from one brain section (**A**). Lesions are identified as dense structures of nuclei stained with Hoechst 33342 (grey) that may be Her2/neu positive (green; parts of B), mKate positive (yellow; parts of D) or sometimes both (overlap green and yellow; E and parts of C). Liposomal DiR fluorescence (red) is also shown, with very limited positivity, highlighted using blue arrows (**B**, **C**, **D**). Scale bars = 150 µm

Representative images of tissue sections from organs of mice with IC JIMT-1^mkate^ tumor implantation are shown in Figs. 6, 7, 8 and 9. Some organs, such as the adrenal gland (Fig. 6A) and ovary (Fig. 6B), are largely overtaken by tumor-growth, with relatively little differentiated tissue remaining (organs from non-tumor bearing animals are shown in the top panels for comparison). Tumor-positive regions within the organs were identified using mKate and Her2/neu expression, as well as histological features as previously described [1]. Accumulation of DiR labeled liposomes is seen in the adrenal gland and ovary, in both the tumor-free and tumor-bearing IC model, without a significant increase in the tumor-bearing animals. Insets show that in fact DiR (red) continues to be present in the non-tumor (mKate and Her2/neu negative) regions of these organs. A representative kidney is also shown (Fig. 6C), where a small mKate and Her2/neu positive tumor nodule is observed, but very little DiR labelled liposome accumulation is noted relative to the rest of the otherwise normal organ where glomeruli are consistently DiR positive.

When JIMT-1^mkate^ cells are inoculated IC, tumor growth is also noted in the liver, lung and brain. Again, the expression of mKate and Her2/neu is variable for all three sites, requiring assessment of both to identify tumor areas in stained cryosections. In the liver of one animal (Fig. 7), DiR is evenly distributed throughout the normal organ, with notably less accumulation in the tumor positive lesions, which appear to largely exclude liposome delivery (Fig. 7B and C). In the lung (Fig. 8) very little DiR is seen in the normal tissue, with no appreciable increase in the tumor lesions identified via Her2/neu and mKate expression (Fig. 8 B, C, D). In the brain (Fig. 9), groups of 10–30 tumor cells were found to localize around select blood vessels located throughout the organ, sometimes alone or sometimes in clusters. Very little DiR is seen in the normal tissue, however, some areas with tumor cell infiltration are positive for DiR while other metastatic sites remain negative. It should be noted that this brain sample was selected for illustration due to the numerous metastatic clusters in the same area that highlight the different patterns of growth. This sample was also used in our previous work examining vasculature (CD31) and Her2/neu distribution [1].

Figures 6, 7, 8 and 9 demonstrate the heterogeneous distribution of DiR labeled liposomes for JIMT-1^mkate^ tumors that arise in multiple sites. JIMT-1^mkate^ tumors in the kidney, adrenal gland and ovary showed little to no DiR fluorescence, while some lesions in the liver, lung and brain exhibited some liposome delivery, though none with significant accumulation. The fact that DiR labelled liposomes can be seen in some brain and lung tumors is surprising considering that in tumor naïve animals, liposomes do not accumulate in the organs at all. This indicates that changes in the vasculature within organs with JIMT-1^mkate^ tumors has occurred, allowing liposome extravasation and some small amount of tissue penetration. No preference for liposomal distribution was uncovered in any particular metastatic site in the IC model.

### Efficacy of doxil and irinophore C in mice bearing JIMT-1 tumors established following orthotopic or intracardiac injection of JIMT-1 cells

**Fig. 10:**
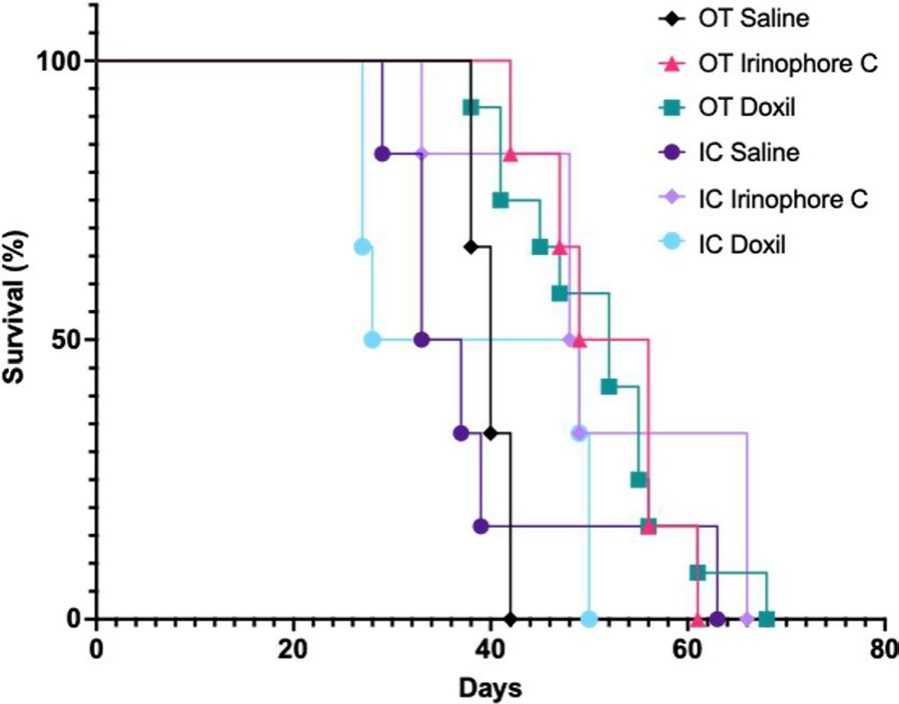
Mice bearing OT and IC JIMT-1^mKate^ tumors were treated with liposomal doxorubicin (Doxil^®^) (1 mg/kg; Q14Dx2), or Irinotecan (Irinophore C) (20 mg/kg; Q4Dx3) such that the final liposomal lipid concentration was 100 mg/kg). Kaplan–Meier curves are shown where mice were euthanized due to tumor progression or poor health status

The efficacy of liposomal anticancer drug formulations was assessed in our model of metastatic disease using Doxil, an approved liposomal doxorubicin formulation^4−6^, and Irinophore C, a liposomal irinotecan formulation that exhibits significant therapeutic effects in multiple tumor models [13–17]. Results are shown for mice with single large OT JIMT-1 tumors and for mice with multiple, smaller IC JIMT-1 tumors in different organs/tissues established following IC injection of the JIMT-1 cells. The liposomal drugs were administered at a liposomal lipid dose of 100 mg/kg to match the liposome dose that was used for the liposome distribution studies described above. This selected lipid dose contained well-tolerated, low doses of irinotecan (20 mg/kg; Q4Dx3) or doxorubicin (1 mg/kg; Q14Dx2). Experimental endpoints were pre-determined and standardized based on health status symptoms suggestive of advanced disease progression, with animals euthanized accordingly. Both Doxil and Irinophore C treatment groups exhibited a delay in OT tumor growth during the treatment period as determined by caliper measurements (data not shown). Kaplan–Meir survival curves were generated based on the day after animals were euthanized due to disease progression. The results have been summarized in Fig. 10. The median survival time (MST) for mice treated with Doxil was 43 and 39 days for mice with JIMT-1 tumors in the OT and IC model respectively. Similarly, the MST for mice treated with Irinophore C was 53 days and 49 days for JIMT-1 OT and IC tumors respectively. For both drugs and in all tumor growth models, survival times improved relative to untreated animals, suggesting the liposomal formulations were able to achieve some tumor control even at the low doses used.

## Discussion

It is often reported that preclinical efficacy studies with anti-cancer drug candidates do not predict outcomes in clinical trials [59, 60]. One possible explanation for this failure in translation is that preclinical models do not sufficiently recapitulate the heterogeneous nature of cancer and do not model advanced disease where tumor growth is occurring in multiple sites. Both intratumor and inter-metastatic heterogeneity are key characteristics of patients who make up the population of people enrolled in clinical trials, and those who require better therapeutic options. When first tested in humans with cancer, new drug candidates and their formulations will be assessed in late-stage patients that have failed standard of care and will have chemotherapy resistant tumors residing in multiple sites. Phase 1 studies are designed to define a safe dose to be administered and will typically not be tumor site specific, while Phase 2 studies will be in a better-defined patient population, but still a population that has metastatic, and likely resistant disease. We suggest that new agents need to be assessed pre-clinically in models that recapitulate complex, advanced cancer. Perhaps too simplistically, it has been argued by our team [1, 49] and others [61, 62] that preclinical tests should be completed in mice with established metastatic disease. These models do not have to mimic the metastatic process, but they should be models where tumors are present in multiple organs and tissues, reflecting the intratumor and inter-metastatic heterogeneity that is seen in the clinic. This type of preclinical modeling, where tumors arise in multiple sites, is very challenging but necessary to close the gap between the bench and the bedside.

While being able to model complex late-stage cancer is necessary, it is not sufficient to define better drug candidates that could improve patient outcomes. Additional attention needs to be paid to drug design and drug delivery. We believe that designing and developing drug delivery systems (DDS) specifically for complex, heterogeneous disease, represents an important area of research with the potential to make significant progress in developing improved cancer drugs. DDSs such as liposomes may help to improve efficacy of conventional drugs and drug candidates by overcoming challenges related to the optimal delivery of the therapeutic(s) to tumors that are heterogeneous and are established in multiple tissues/organs. Many examples of liposomal drug formulations that improve efficacy over free drugs can be found in the literature [63]. The mechanism through which improved efficacy occurs has often been attributed to the EPR effect [10–17], however, additional mechanisms have been suggested to have greater influence on activity of liposomal formulations in vivo [27–30]. Determining how drug carriers improve drug activity is critical in designing more effective DDSs.

In the current study, our research team sought to explore the delivery of liposomes in the context of tumors arising in different tissues/organs following inoculation of a defined cell line. In mice that do not have tumors, we demonstrate that the distribution of liposomes is, unsurprisingly, heterogeneous. Liposomes are eliminated from the blood compartment over time and accumulate in the liver. Perhaps surprisingly, there appears to be localization of DiR labeled liposomes in the adrenal gland as measured by fluorescent imaging as well as liposome associated [3H]-CHE levels. Previous studies have suggested that liposomal formulations can localize to the adrenal gland [63] and investigators have even designed liposomal drug formulations for the treatment of neuroblastoma in this organ [64]. Given the role of the adrenal gland in regulating metabolism and the immune system, this information may be helpful in guiding the development of future liposomal formulations.

Both OT and systemic tumors were established using an isogenic cell line (JIMT-1^mKate^). Our results demonstrate that liposomes do accumulate in OT tumors over time, though the microregional distribution within the tumors is limited. Significant inter-vessel heterogeneity is observed. Most vessels are negative for DiR signal, and where DiR fluorescence is observed overlapping with blood vessels, the signal remains within a few µm distance, suggesting very little extravascular distribution. These findings were replicated in two additional OT models established using MDA MB 231 and MDA MB 435/LCC6 cell lines. In both cases, inter-vessel heterogeneity is seen with very little accumulation of DiR in the tumors themselves (data not shown). Other reports examining accumulation of large nanoparticle therapeutics in solid tumor models have shown similar results [32, 40].

The IC JIMT-1^mKate^ model where tumors arise in different tissues/organs is not a model of the metastatic process where systemic disease arises following growth and perhaps removal of an orthotopic tumor. However, upon intracardiac inoculation, cells seed in multiple organ sites within the same animal resulting in measurable tumors. Using this model, we were able to establish disease loci with considerable intratumor and inter-metastatic heterogeneity of the tumor microenvironment. This heterogeneity exists even though the tumors that arise following IC injection are from an isogenic JIMT-1^mKate^ cell line. Each organ has an environment that supports the development of tumors with unique characteristics that impact lesion size, vascular pattern and function, and the degree to which normal organ function may become compromised. We show that vascular structures within tumor lesions typically do not match the microenvironment of the surrounding normal tissue. Any of these features may in turn influence the accumulation of therapeutics and nanoparticles such as liposomes. Our data suggests that DiR labeled liposomes can accumulate more readily in normal tissue, such as the liver, than in tumors even when those tumors grow within the liver. If the goal is to maximize liposome delivery to the tumor and minimize delivery to normal tissue, this result is exactly what needs to be avoided.

In previous studies, our team provided evidence to show that docetaxel (a non-liposomal drug) was less efficacious in an IC breast tumor model compared to both a solid OT and ascites breast tumor model [49]. This study provided evidence that free drugs have differential effects depending on the model used, and the results exemplified the need to evaluate the activity of drugs/drug candidates in models with multiple sites of established tumors prior to committing resources to develop a drug for clinical trials. More importantly, these results suggested that there is a need for DDSs to overcome these challenges. Our group as well as others have demonstrated that liposomal drug formulations are indeed better than free drugs at eliciting a response in solid tumors [40, 65]. For example, Ngai et al. showed that Doxil, a liposomal doxorubicin formulation prepared with PEG-modified lipids, increased the spatial distribution of cell death as measured by caspase 3 activation in 4T1 breast tumors compared to what can be achieved with free doxorubicin [65]. In another example, we have shown that there is limited accumulation of the liposomal formulation of irinotecan, Irinophore C, in solid tumors relative to that of the free drug, which is distributed widely through the tissues before rapidly washing out. Despite this finding, the liposomal formulations were shown to be far superior in arresting proliferation in all areas of the tumor, even those without accumulated liposomes. Further, Irinophore C had an overall greater anti-cancer effect [40]. This evidence in solid tumors supports the idea that the EPR effect is not necessary for activity of liposomal drugs and therefore prompted the question of whether or not EPR was relevant in the context of metastatic disease. When initiating the studies described in this report, our hypothesis was that liposomal drug formulations would be less effective when used to treat a tumor model where the EPR effect was less apparent, such as in metastatic sites known to have limited vasculature. However, this was not the case for the two liposomal drugs tested (Doxil and Irinophore C). Both drugs were effective in mice with either OT tumors or IC tumors growing in multiple sites despite evidence of inconsistent or even a lack of accumulation of liposomes.

## Conclusions

The data reported can help to inform the design of better liposomal drug formulations and the design of delivery systems, in general. We conclude that the EPR effect is less important in dictating the activity of liposomal drugs. It is more likely that the rate at which associated drugs are released from liposomes within the blood compartment is playing a larger role in the improvement of efficacy seen with liposomal drugs. As demonstrated by Nguyen et al. less than 5% of injected nanoparticles reach tumor tissues, supporting the notion that passive accumulation via EPR might be less impactful than previously thought [67]. The ability of Irinophore C to achieve vascular normalization [16] and efficacy against glioblastoma [12] despite the fact that liposomes do not cross normal blood vessel structures or the blood brain barrier suggests mechanisms beyond passive liposomal delivery. Therefore, more attention should be paid to designing liposomes to control the rate of liposome elimination from the blood compartment over time, as well as the rate at which the payload leaves the liposomes within the blood compartment. Well-designed drug carriers should help to prolong the plasma half-life of the active agent(s) as well as regulate the rate that the active agent(s) are released from the carrier. The effect may mimic metronomic dosing of chemotherapy, allowing for low levels of continuous exposure to the agent(s) of interest [66]. Furthermore, the type of phenotypic heterogeneity described in the current paper remains best studied using an observational and qualitative approach at this time. Our hope is that with advances in machine learning, a high throughput analytical process and appropriate statistical methodology based on principles of landscape metrics might be developed to do a more quantitative and statistically relevant examination of heterogeneity in the near future.

## Data Availability

The datasets during and/or analysed during the current study available from the corresponding author on reasonable request.

## Abbreviations

TME: Tumor microenvironment
OT: Orthotopic
IC: Intracardiac
mIHC: Multiplex immunohistochemistry
EPR: Enhanced permeability and retention
DDS: Drug delivery systems
FBS: Fetal bovine serum
DSPC: 1,2-Distearoyl-sn-glycero-3-phosphocholine
DiR; DiC_18_(7): 1,1 Dioctadecyl-3,3,3′,3,′-Tetramethylindotricarbocyantine Iodide
LSC: Liquid scintillation counting
IVFI: In vivo Fluorescence imaging
AUC: Area under the curve
MST: Median survival times

## Declarations

### Patents

Patents protecting Irinophore C reached the international stage of filing, but these patents have since been abandoned.
